# Supplementary material for: New TEMPO–Appended 2,2′-Bipyridine-Based Eu(III), Tb(III), Gd(III) and Sm(III) Complexes: Synthesis, Photophysical Studies and Testing Photoluminescence-Based Bioimaging Abilities
Source: Molecules. 2022 Dec 1;27(23):8414. doi: 10.3390/molecules27238414 (PMC9739109; doi:10.3390/molecules27238414)

New TEMPO–Appended 2,2′–Bipyridine-Based Eu(III), Tb(III), Gd(III) and Sm(III) Complexes: Synthesis, Photophysical Studies and Testing Photoluminescence-Based Bioimaging Abilities

Nataliya V. Slovesnova [1,2,3], Artem S. Minin [1,3,4], Anna V. Belousova [5], Aleksey A. Ustyugov [6], Kirill D. Chaprov [6], Alexey P. Krinochkin [1,3], Maria I. Valieva [1,3], Yaroslav K. Shtaitz [1], Ekaterina S. Starnovskaya [1,3], Igor L. Nikonov [1,3], Anton N. Tsmokalyuk [1], Grigory A. Kim [1,3], Sougata Santra [1], Dmitry S. Kopchuk [1,3], Emiliya V. Nosova [1,3,*] and Grigory V. Zyryanov [1,3]

[1] Ural Federal University, 19 Mira Street, 620002 Yekaterinburg, Russia
[2] Urals State Medical University, 3 Repina Street, 620028 Yekaterinburg, Russia
[3] I. Ya. Postovskiy Institute of Organic Synthesis, UB of the RAS 22, S. Kovalevskoy Street, 620219 Yekaterinburg, Russia
[4] M.N. Miheev Institute of Metal Physics, Ural Branch of the RAS, Russian Federation 18, S. Kovalevskoy Street, 620108 Yekaterinburg, Russia
[5] Institute of Immunology and Physiology, Ural Branch of the RAS 106, Pervomaiskaya Street, 620049 Yekaterinburg, Russia
[6] Institute of Physiologically Active Compounds at Federal Research Center of Problems of Chemical Physics and Medicinal Chemistry of the RAS 1, Severniy Proezd, 142432 Chernogolovka, Russia
* Correspondence: e.v.nosova@urfu.ru

Supporting information

Methods

All reagents were purchased from commercial sources and used without further purification. Silica gel 60 (Kieselgel 60, 230-400 mesh) was used for the column chromatography. NMR spectra were recorded on a Bruker Avance (600 MHz and 400 MHz) spectrometers, 298 K, digital resolution ± 0.01 ppm, using TMS as internal standard. Mass spectra were recorded on a MicrOTOF-Q II mass spectrometer (Bruker Daltonics) with electrospray ionization. Elemental analyses were performed on a PE 2400 II CHN-analyzer (Perkin Elmer). The ESR spectra of complexes were recorded at room temperature using a Bruker ELEXSYS E 500 CW-EPR spectrometer operating at the X-band frequency. Modulation amplitude 0.1mT, power is 2.000 mW, 1024 points.

Starting isonitrosoacetophenone hydrazone **2** [S1], 1,2,4-triazino-3-oxide **1** and 5-cyano-1,2,4-triazine **4** [S2] were synthesized according to previously described methods. Other reagents are commercially available.

Synthetic procedures

Methyl 6-(3-cyano-4-phenyl-6,7-dihydro-5*H*-cyclopenta[*c*]pyridin-1-yl)nicotinate (5). To a solution of 1,2,4-triazine **4** (310 mg, 0.98 mmol) in toluene (10 ml) was added 1 equiv. morpholinecyclopentene (150 mg, 0.48 mmol). The resulting mixture was stirred at reflux in argon atmosphere for 2 h, after which another 1 equiv. morpholinecyclopentene and stirred at reflux for 1 h. After completion, the solvent was removed under reduced pressure, glacial acetic acid (3 ml) was added to the residue and brought to a boil 3 times, after which the reaction mixture was suspended in ethanol (5 ml) and filtered. Yield 305 mg (0.86 mmol, 88 %), ^{1}H NMR (400 MHz, DMSO-d_6), δ: 2.04 – 2.12 (2H, m CH_2), 2.90 (2H, t, ^{3}J = 7.2 Hz, CH_2), 3.54 (2H, t, ^{3}J = 7.2 Hz, CH_2), 3.94 (3H, s, Me), 7.53 – 7.61 (5H, m, Ph), 8.42 – 8.46 (1H, m, H-2(Py)), 8.47 – 8.51 (m, 1H, H-6(Py)), 9.22 (1H, d, ^{4}J = 2.0 Hz, H-5(Py)). ESI-MS *m/z*: 356.14 [M+H]. Molecular formula $C_{22}H_{17}N_3O_2$ Elemental analysis: found C, 74.63; H, 4.68 ; N, 11.69 %; requires C, 74.35; H, 4.82; N, 11.82 %.

1-(5-(Hydroxymethyl)pyridin-2-yl)-4-phenyl-6,7-dihydro-5*H*-cyclopenta[*c*]pyridine-3-carbonitrile (6). The starting compound **5** (350 mg, 0.98 mmol) was dissolved in ethanol-chloroform 8:1 (45 ml) and followed by 5 equiv. sodium borohydride (186 mg, 4.92 mmol) was added. The resulting mixture was refluxed for 8 h, after which the solvent was removed under reduced pressure. The residue was suspended in EtOH (30 ml), 5 equiv. sodium borohydride added

again and the reaction mixture was refluxed for 8 hours yet. After completion, 30 ml of water was added to the reaction mass and the reaction product was extracted into DCM (3 times 25 ml). The resulting reaction product solution was dried over anhydrous sodium sulfate, and the solvent was removed under reduced pressure. Yield 217 mg (0.66 mmol, 67 %). ^{1}H NMR (500 MHz, $CDCl_3$), δ: 2.09 – 2.15 (2H, m, CH_2), 2.90 (2H, t, ^3J = 7.5 Hz, CH_2), 3.58 (2H, t, ^3J = 7.5 Hz, CH_2), 4.83 (2H, s, $\underline{CH_2}$OH), 7.45 – 7.54 (5H, m, Ph), 7.87 (1H, dd, ^3J = 8.0 Hz, ^4J = 2.0 Hz, H-4(Py)), 8.35 (1H, d, ^3J = 8.0 Hz, H-3(Py)), 8.69 (1H, d, ^4J = 2.0 Hz, H-6(Py)). ^{13}C NMR (100.61 MHz, $CDCl_3$), δ: 24.9, 32.5, 34.2, 62.6, 117.5, 123.2, 128.8(2C), 129.0(2C), 129.1, 129.9, 134.6, 135.4, 136.1, 138.3, 143.5, 147.4, 152.0, 155.8, 155.8. ESI-MS *m/z*: 328.15 [M+H]. Molecular formula $C_{21}H_{17}N_3O$ Elemental analysis: found C, 76.93; H, 5.28; N, 12.90%; requires C, 77.04; H, 5.23; N, 12.84%.

1-(5-Formylpyridin-2-yl)-4-phenyl-6,7-dihydro-5*H*-cyclopenta[*c*]pyridine-3-carbonitrile (7). To a solution of starting compound **6** (210 mg, 0.64 mmol) in dichloroethane (40 ml) was added 10 eq. manganese oxide (558 mg, 6.40 mmol). The resulting mixture was stirred at 65°C for 10 h. Then another 5 equiv. manganese oxide (279 mg, 3.2 mmol) was added and reaction mass was stirred again at 65°C for 8 h. After completion, the reaction mixture was filtered using a Schott filter, after which the residue was purified by flash chromatography (DCM:AcOEt 9:1 system) and the solvent was removed under reduced pressure. Yield 113 mg (0.35 mmol, 54 %). ^{1}H NMR (400 MHz, $CDCl_3$), δ: 2.11 – 2.19 (2H, m, CH_2), 2.93 (2H, t, ^3J = 7.6 Hz, CH_2), 3.65 (2H, t, ^3J = 7.6 Hz, CH_2), 7.45 – 7.56 (5H, m, Ph), 8.31 (1H, dd, ^3J = 8.0 Hz, ^4J = 2.0 Hz, H-4(Py)), 8.61 (1H, d, ^3J = 8.0 Hz, H-3(Py)), 9.14 – 9.17 (1H, m, H-6(Py)), 10.19 (1H, s, HC=O). ^{13}C NMR (100.61 MHz, $CDCl_3$), δ: 24.9, 32.5, 34.6, 117.2, 123.5, 128.9(2C), 129.0(2C), 129.3, 130.2, 130.6, 134.3, 136.6, 139.0, 144.7, 150.5, 150.9, 156.3, 161.1, 190.6. ESI-MS *m/z*: 326.13 [M+H]. Molecular formula $C_{21}H_{15}N_3O$ Elemental analysis: found C, 77.38; H, 4.72; N, 12.98%; requires C, 77.52; H, 4.65; N, 12.91%.

(*Z*)-4-(((6-(3-cyano-4-phenyl-6,7-dihydro-5*H*-cyclopenta[*c*]pyridin-1-yl)pyridin-3-yl)methylene)amino)-2,2,6,6-tetramethylpiperidin-1-olate (8). To a solution of compound **7** (112 mg, 0.34 mmol) in ethanol (10 ml) was added TEMPO (59 mg, 0.34 mmol) and the reaction mixture was stirred at 60°C in an Ar atmosphere for 6 h, after which the solvent was removed under reduced pressure. Yield 158 mg (0.33 mmol, 96 %). ESR-spectra (mT): g_i = 2.00732, $a^1{}_N$ = 1.571, $a^2{}_N$ = 1.582, LWPP = 0.180 (N-O•). ESI-MS *m/z*: 478.26 [M+]. Molecular formula $C_{30}H_{32}N_5O$ Elemental analysis: found C, 75.32; H, 6.86; N, 14.48 %; requires C, 75.29; H, 6.74; N, 14.63 %.

CuCl•(*Z*)-4-(((6-(3-carboxy-4-phenyl-6,7-dihydro-5*H*-cyclopenta[*c*]pyridin-1-yl)pyridin-3-yl)methylene)amino)-2,2,6,6-tetramethylpiperidin-1-olate (9). To a solution of compound **8** (75 mg, 0.16 mmol) in ethanol (10 ml) was added a solution of copper chloride (32 mg, 0.19 mmol) in distilled water (1 mL) and stirred at reflux for 8 h. After completion, ethanol was removed from under reduced pressure, and the resulting residue was filtered off and the precipitate was washed with an excess of distilled water. Yield 61 mg (0.09 mmol, 65 %). ESR-spectra (mT): g_i = 2.00686, $a^1{}_N$ = 1.659, $a^2{}_N$ = 1.660, LWPP = 0.294 (N-O•). ESI-MS *m/z*: 559.20 [M+]. Molecular formula $C_{30}H_{32}N_4O_3CuCl$. Elemental analysis: found C, 60.46; H, 5.39; N, 9.47 %; requires C, 60.50; H, 5.42; N, 9.41 %.

*General method for the synthesis of the Ln(III) complexes **10**.*

Ln•(9)$_3$ (10a-d). To a suspension of copper complex **9** (27 mg, 0.05 mmol) in 20 ml of water:ethanol was added a solution of potassium hydroxide (25 mg, 0.45 mmol) and acetone cyanohydrin (0.04 mL, 39 mg, 0.45 mmol) in 10 ml of water. The resulting mixture was stirred at 50°C for 30 min, then cooled to room temperature, the corresponding $LnCl_3•6H_2O$ salt (6 mg, 0.02 mmol) was added and stirred for 15 min. Next, the reaction product was extracted from the reaction mixture into dichloromethane, the resulting solution was dried over anhydrous sodium sulfate, and the solvent was removed under reduced pressure.

Eu•(9)$_3$. (10a) Yield 14 mg (0.009 mmol, 56 %). ESR-spectra (mT): g_i = 2.00714, $a^1{}_N$ = 1.701, $a^2{}_N$ = 1.662, LWPP = 0.216 (N-O•). ESI-MS *m/z*: 1642.46 [M+H]. Molecular formula $C_{90}H_{96}N_{12}O_9Eu$. Elemental analysis: found C, 65.96; H, 5.69; N, 10.32 %; requires C, 65.84; H, 5.89; N, 10.24 %.

Gd•(9)$_3$. (10b) Yield 15 mg (0.009 mmol 60 %). ESR-spectra (mT): g_i = 2.00718, $a^1{}_N$ = 1.696, $a^2{}_N$ = 1.693, LWPP = 0.199 (N-O•). ESI-MS *m/z*: 1647.67 [M+H]. Molecular formula $C_{90}H_{96}N_{12}O_9Gd$. Elemental analysis: found C, 65.66; H, 5.96; N, 10.09 %; requires C, 65.63; H, 5.88; N, 10.20 %.

Sm•(9)$_3$. (10c) Yield 13 mg (0.008 mmol, 52 %). ESR-spectra (mT): g_i = 2.00717, $a^1{}_N$ = 1.692, $a^2{}_N$ = 1.694, LWPP = 0.197 (N-O•). ESI-MS *m/z*: 1641.46 [M+H]. $C_{90}H_{96}N_{12}O_9Sm$. Elemental analysis: found C, 66.05; H, 5.77; N, 10.23 %; requires C, 65.91; H, 5.90; N, 10.25 %.

Tb•(9)$_3$. (10d) Yield 27 mg (0.007 mmol 48 %). ESR-spectra (mT): g_i = 2.00717, $a^1{}_N$ = 1.683, $a^2{}_N$ = 1.687, LWPP = 0.198 (N-O•). ESI-MS *m/z*: 1648.68 [M+H]. $C_{90}H_{96}N_{12}O_9Tb$. Elemental analysis: found C, 65.81; H, 5.71; N, 10.11 %; requires C, 65.56; H, 5.87; N, 10.19 %.

Literature

[S1]. Dey, B.B. C.—Hydrazoximes of methyl- and phenyl-glyoxals. *J. Chem. Soc. Trans.* **1914**, *105,* 1039–1046. DOI: https://doi.org/10.1039/CT9140501039.

[S2]. Krinochkin, A.P.; Kopchuk, D.S.; Chepchugov, N.V.; Kovalev, I.S.; Zyryanov, G.V.; Rusinov, V.L.; Chupakhin, O.N. Effect of substituent in pyridine-2-carbaldehydes on their heterocyclization to 1,2,4-triazines and 1,2,4-triazine 4-oxides. *Russ. J. Org. Chem.* **2017**, *53*, 963–970. https://doi.org/10.1134/S1070428017070016.

Figure S1. ^{1}H NMR Spectra of compound **5**

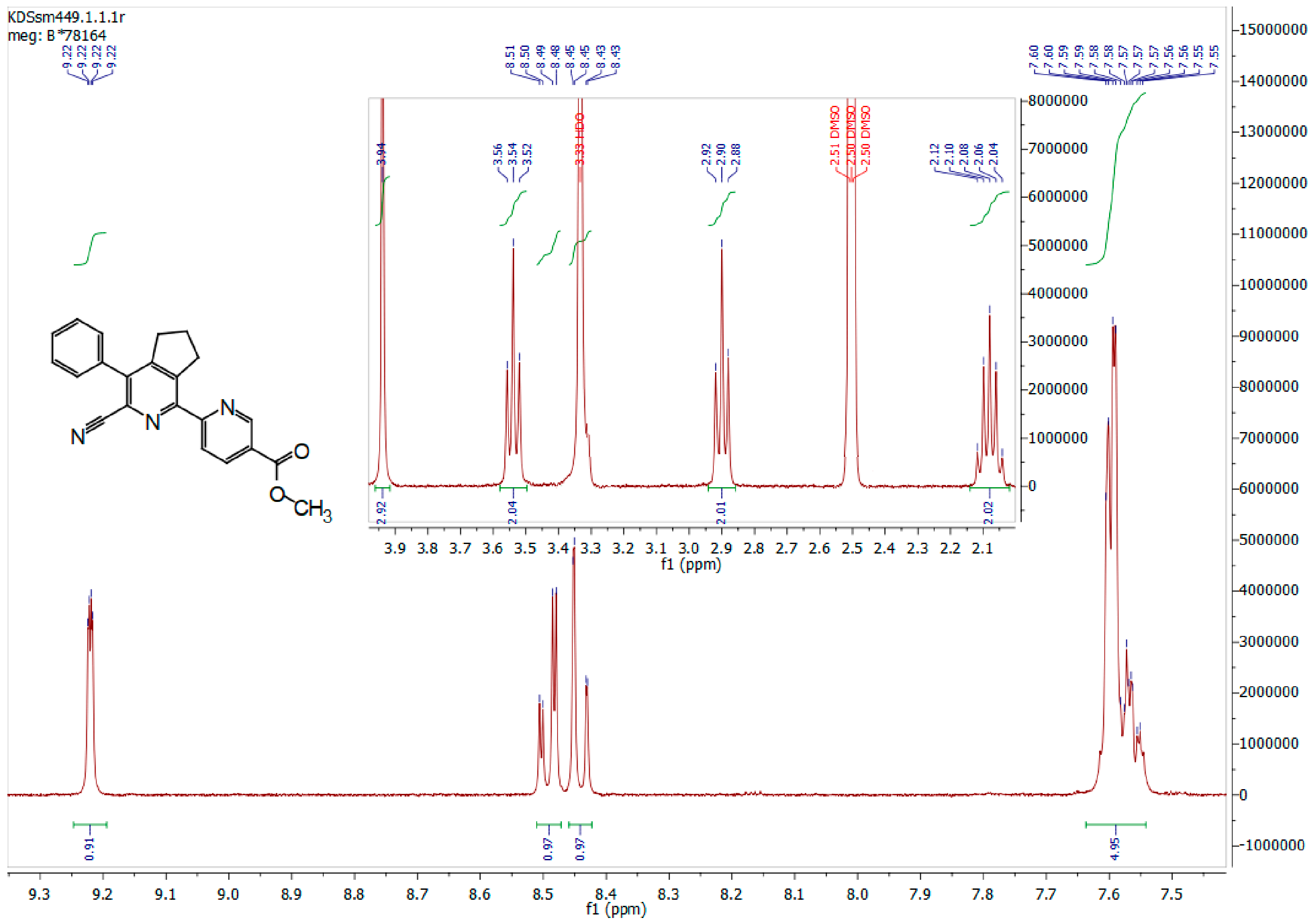

Figure S2. ^{1}H NMR Spectra of compound **6**

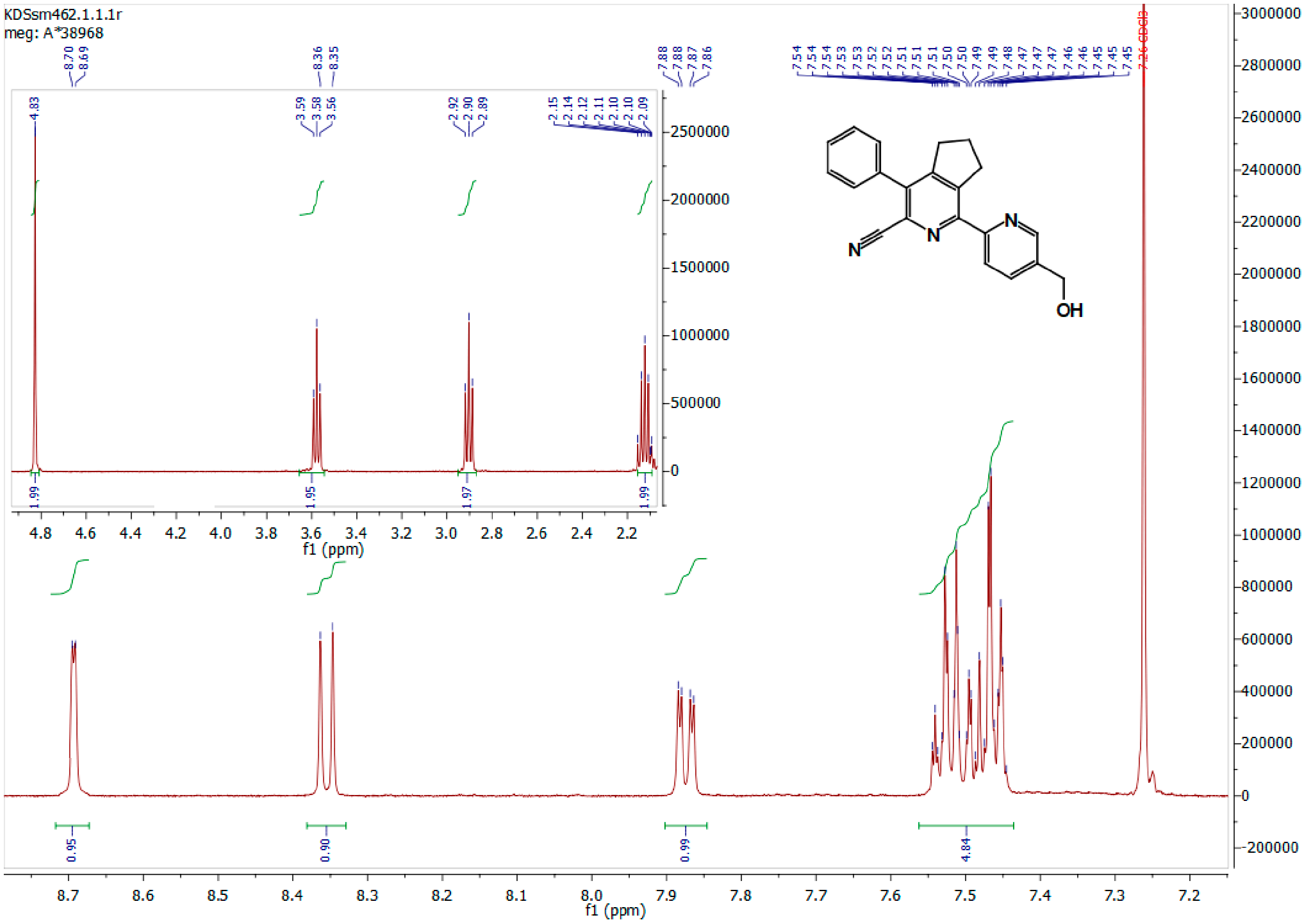

Figure S3 ^{13}C NMR Spectra of compound **6**

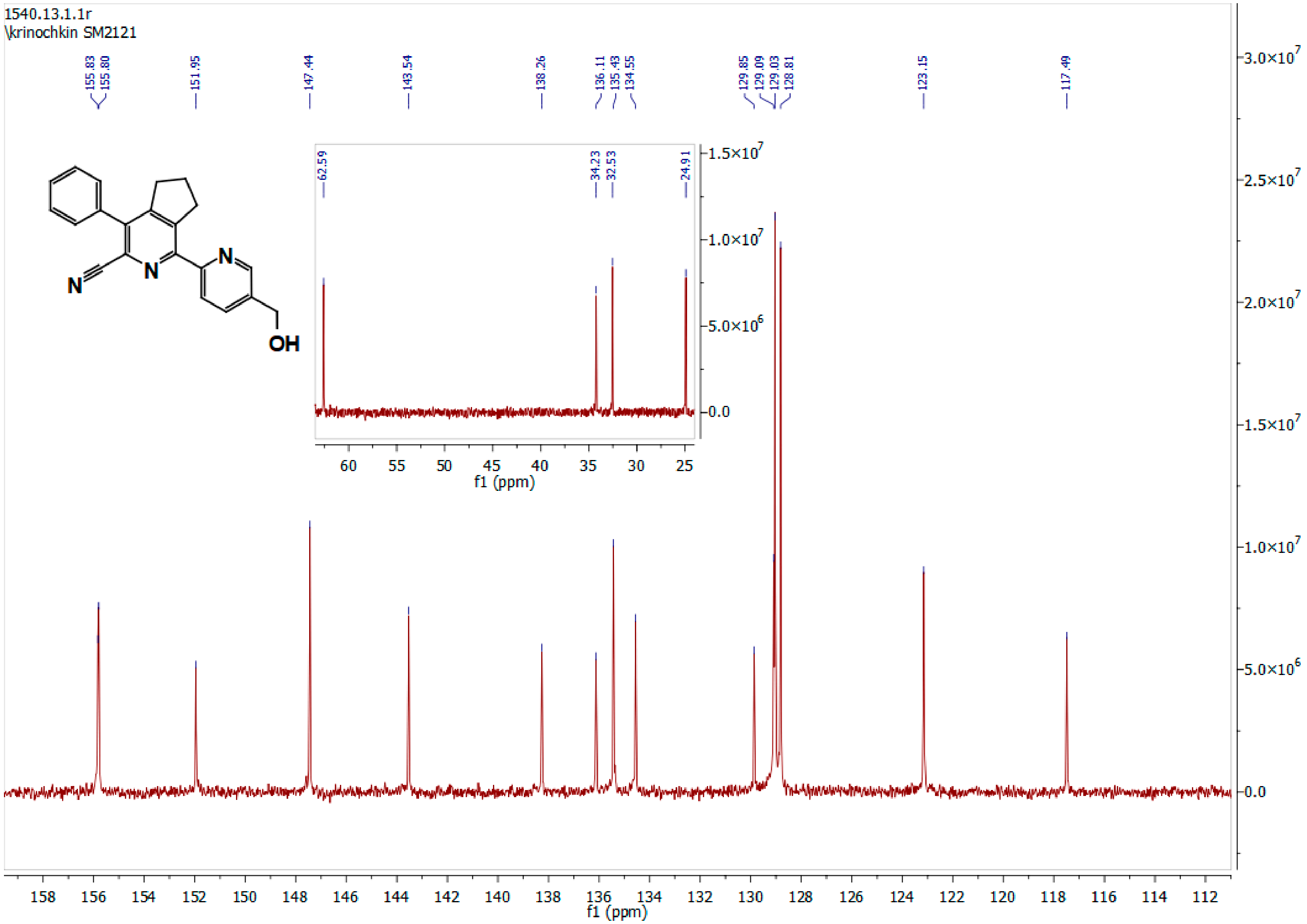

Figure S4. ^{1}H NMR Spectra of compound **7**

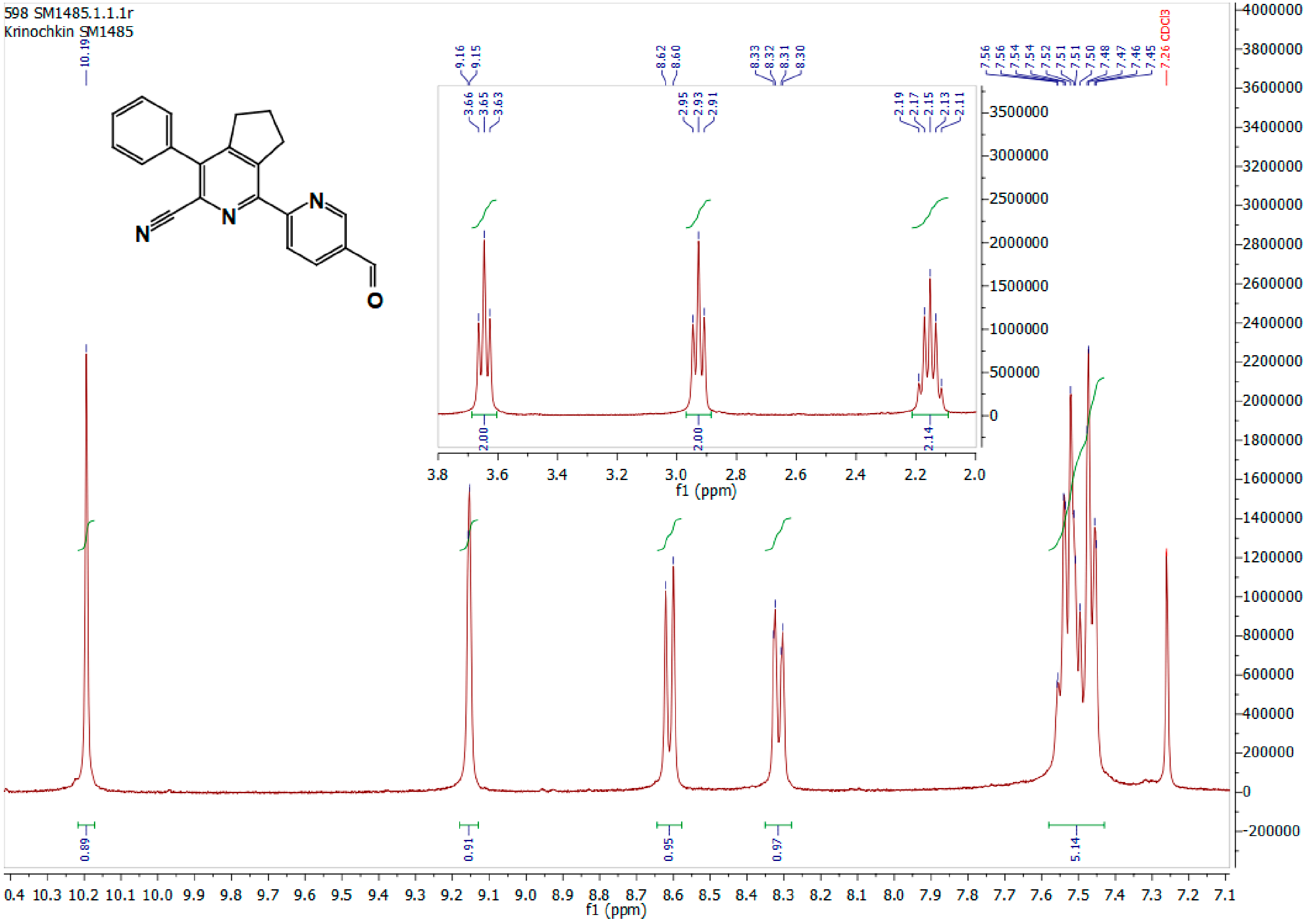

Figure S5. ^{13}C NMR Spectra of compound **7**

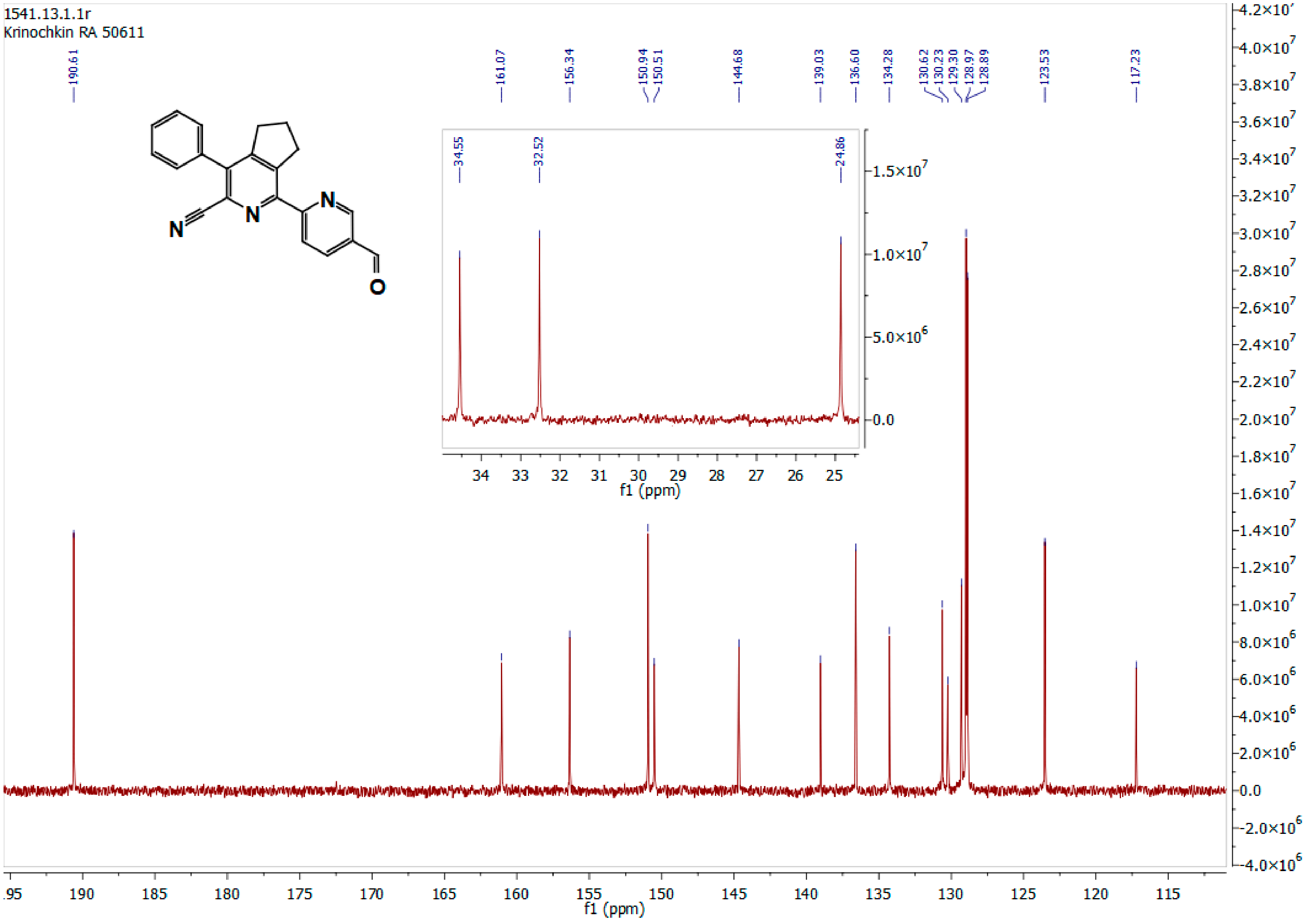

Figure S6. ESR Spectra of compound **8**

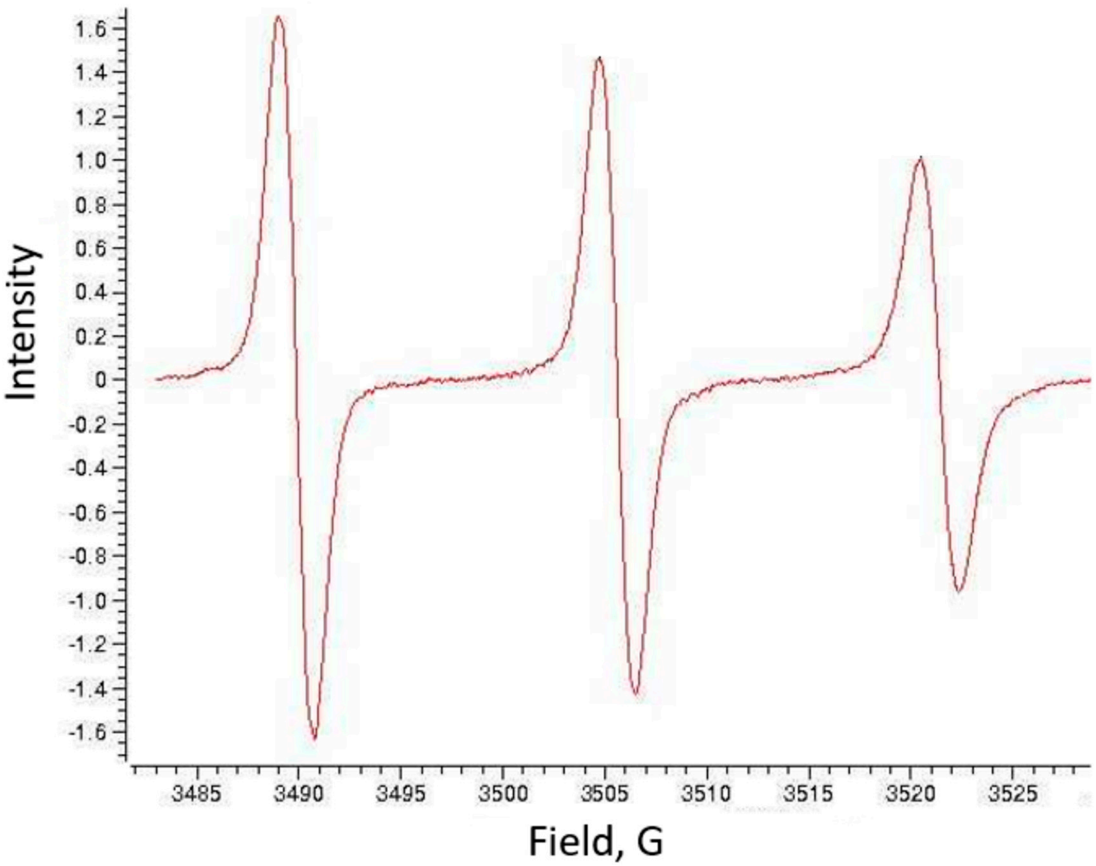

Figure S7. Mass spectra ESI-MS of compound **8**

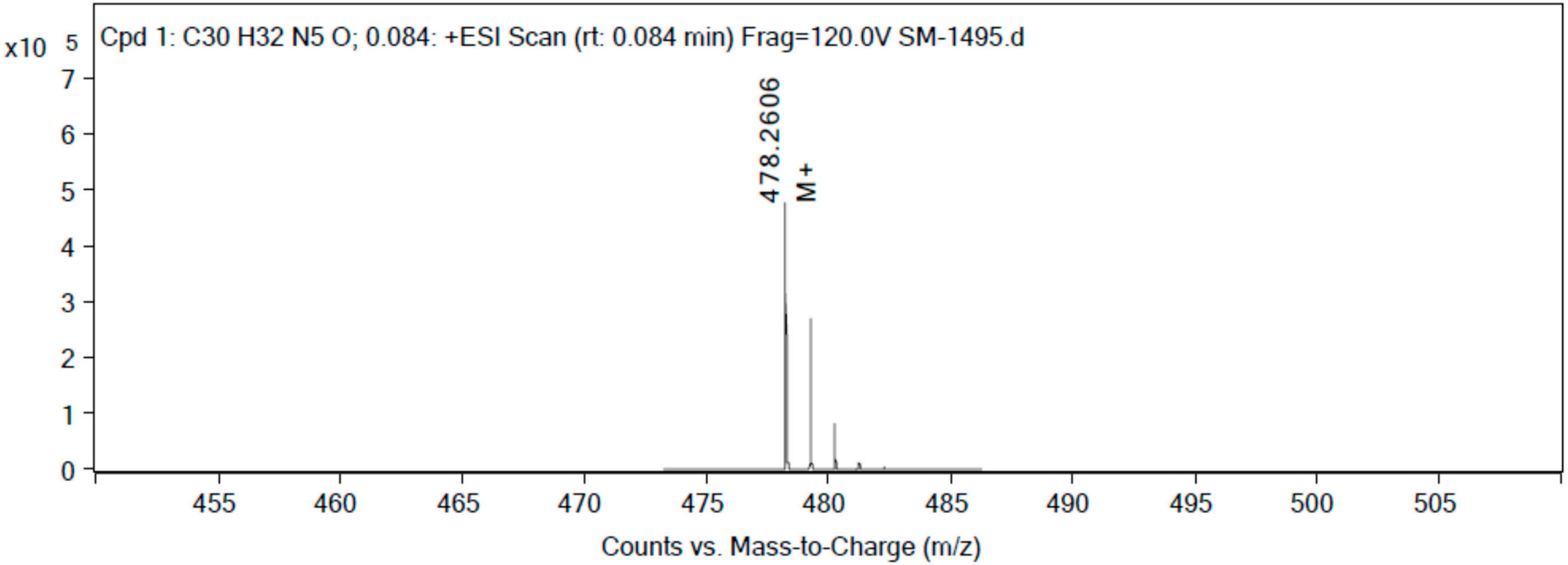

Figure S8. ESR Spectra of compound **9**

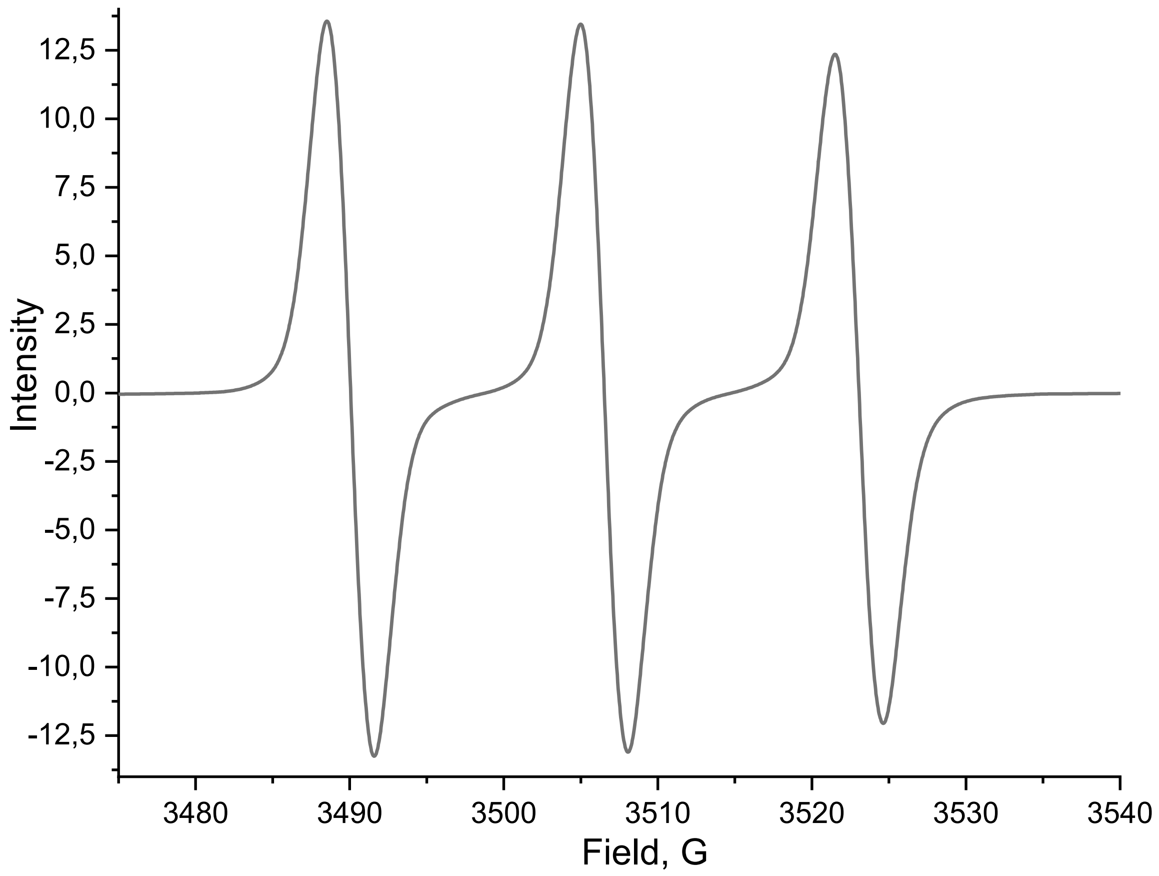

Figure S9. Mass spectra ESI-MS of compound **9**

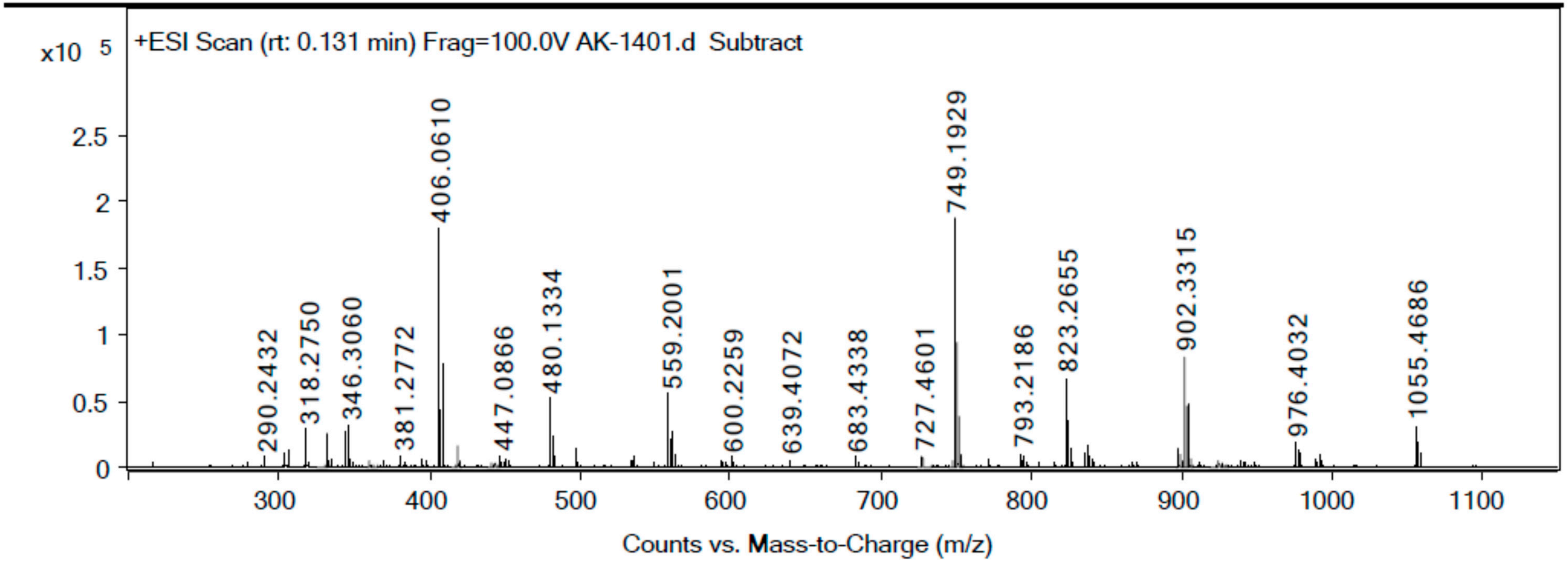

Figure S10. ESR Spectra of compound **10a**

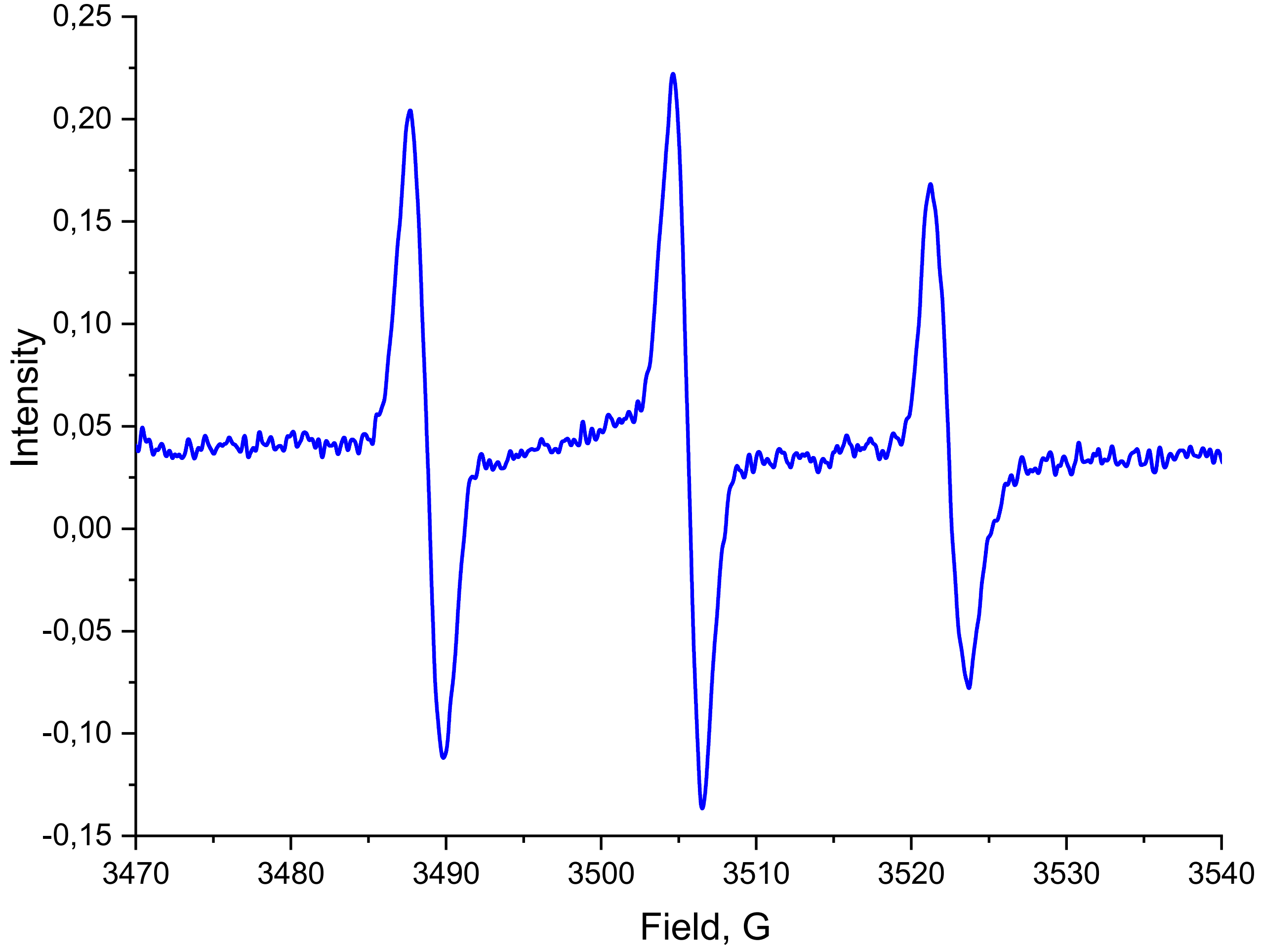

Figure S11. Mass spectra ESI-MS of compound **10a**

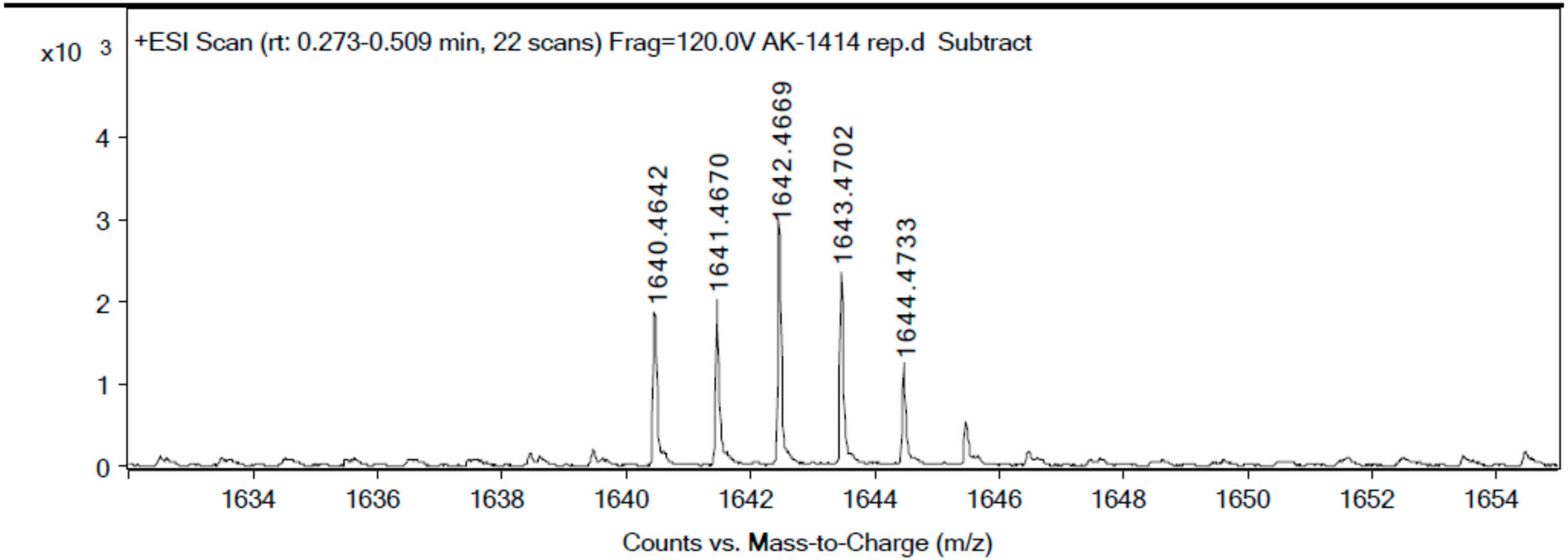

Figure S12. ESR-spectra of compound **10b**

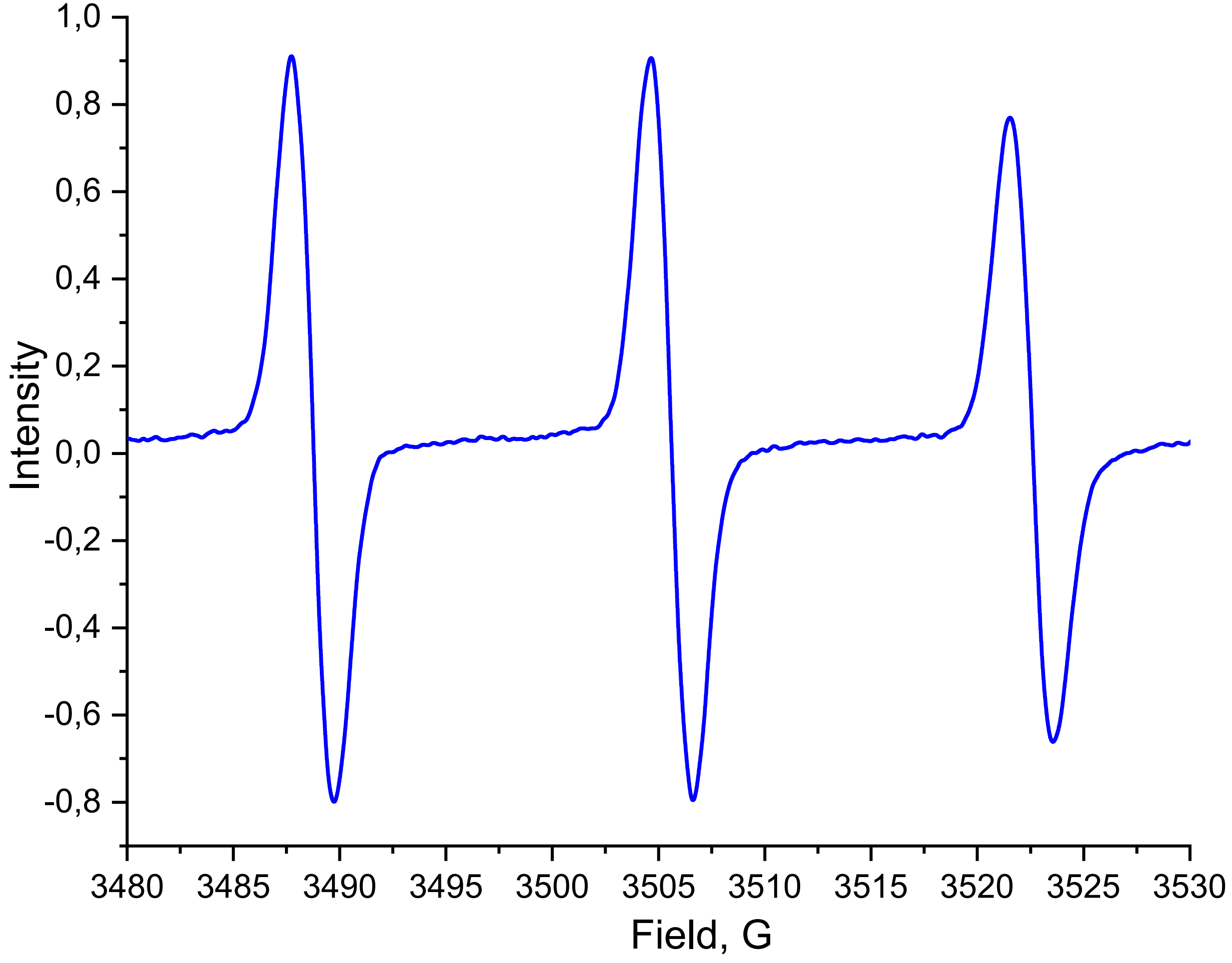

Figure S13. ESR-spectra of compound **10c**

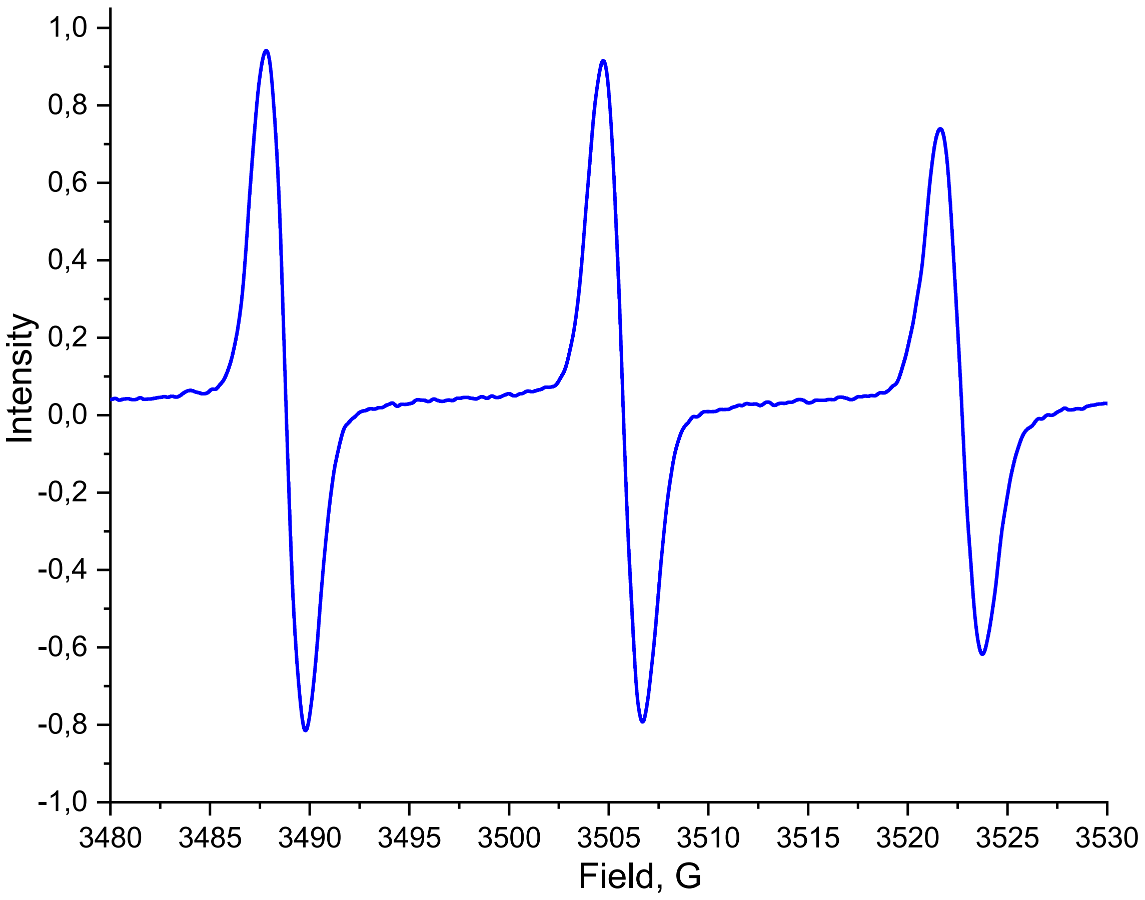

Figure S14. Mass spectra ESI-MS of compound **10c**

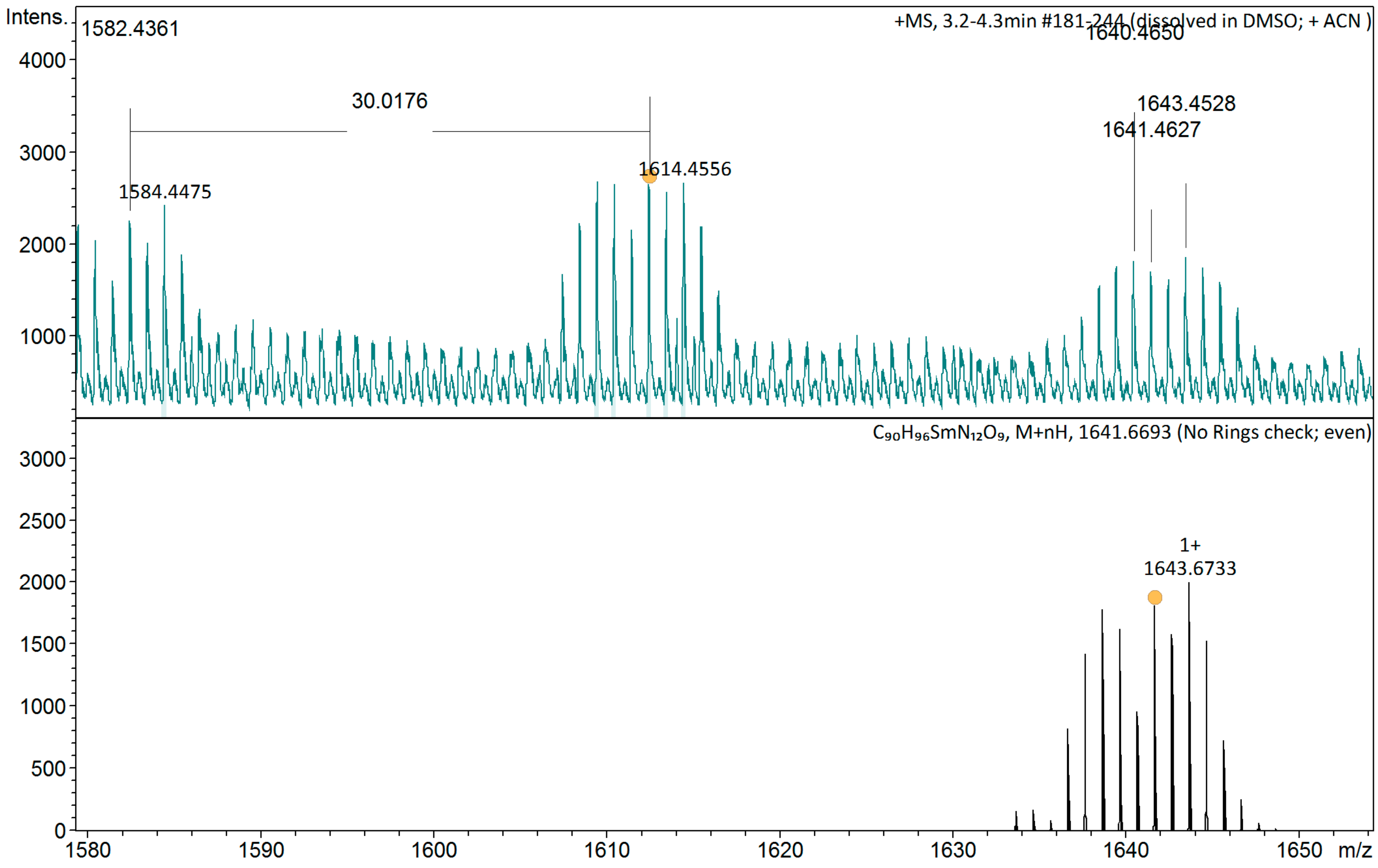

Figure S15. ESR-spectra of compound **10d**

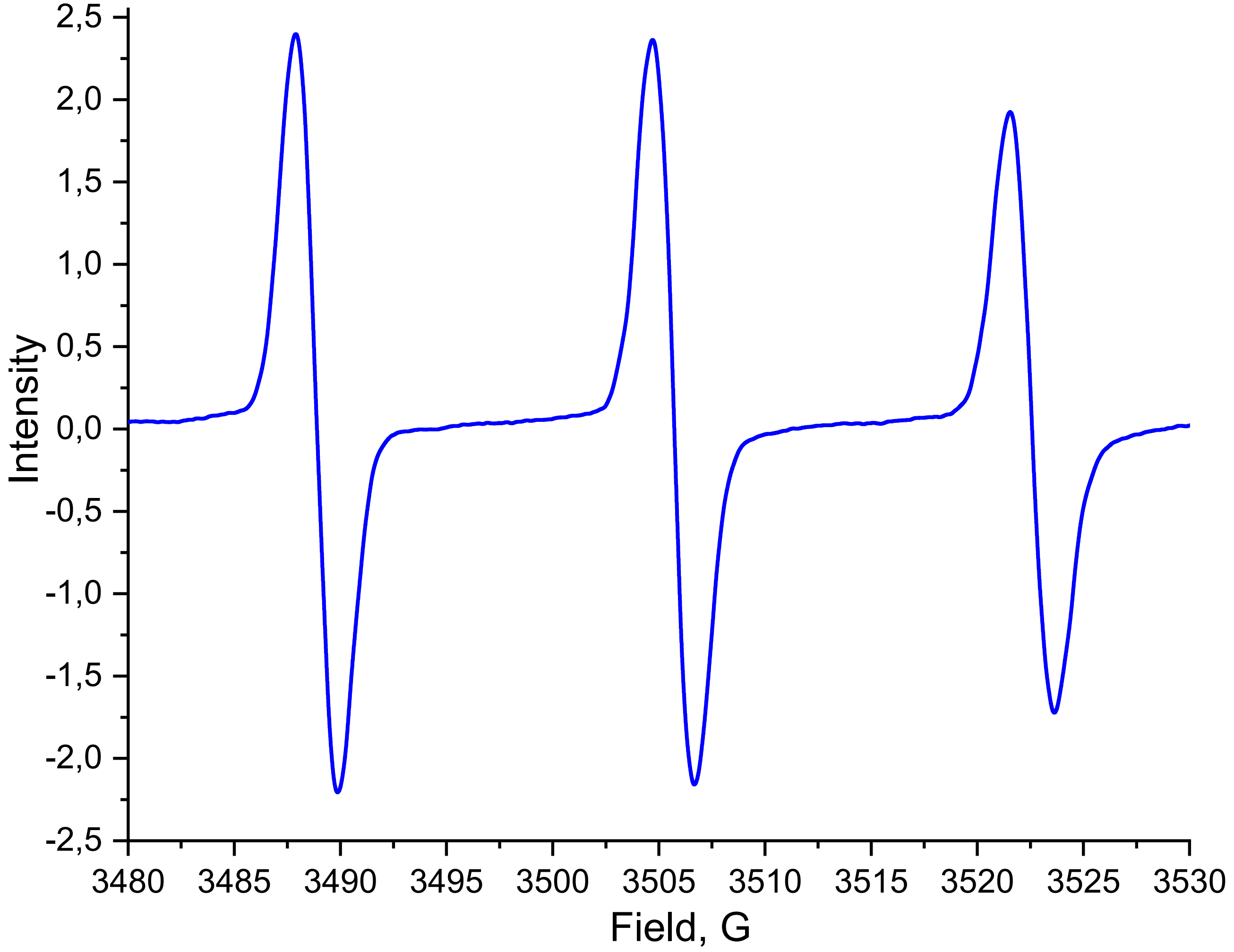

Figure S16. ESR Spectra of Cu(II) signal cation in compound **9**

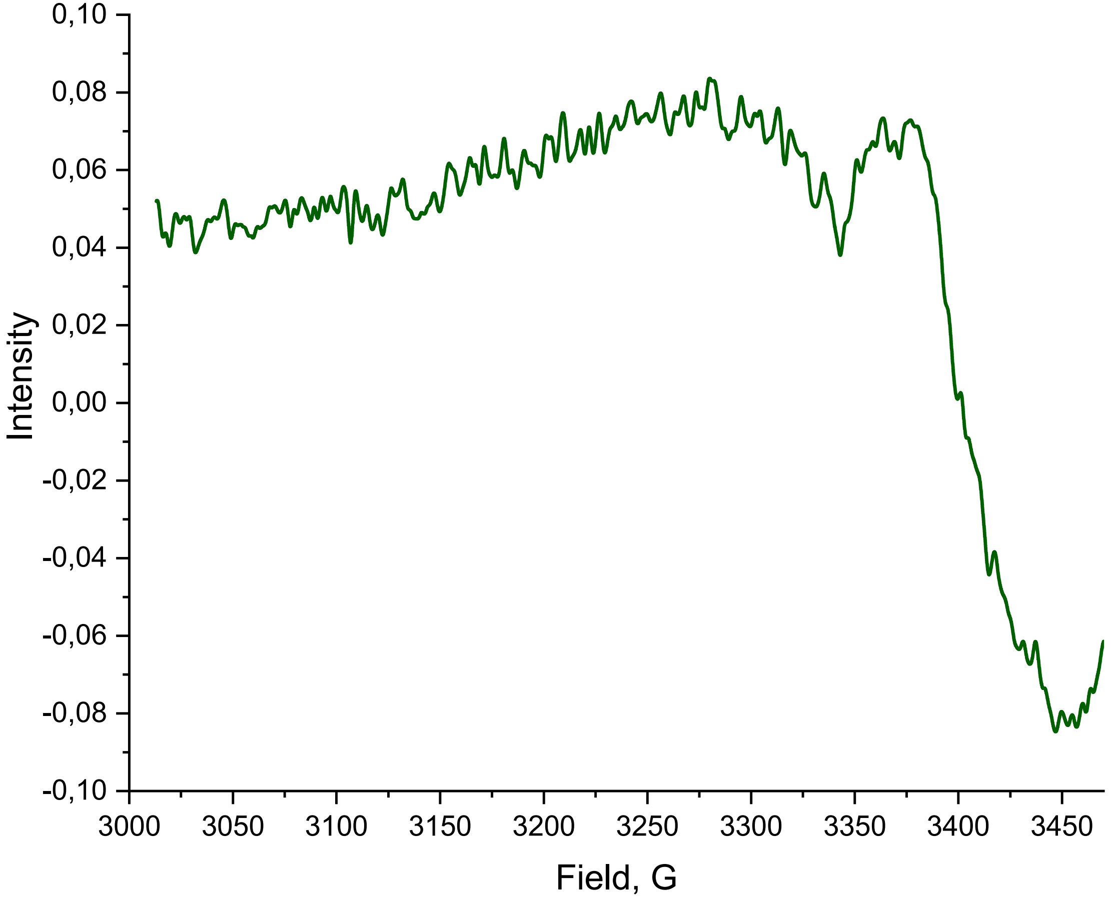

Figure S17. ESR Spectra of compound **10a** in THF over time

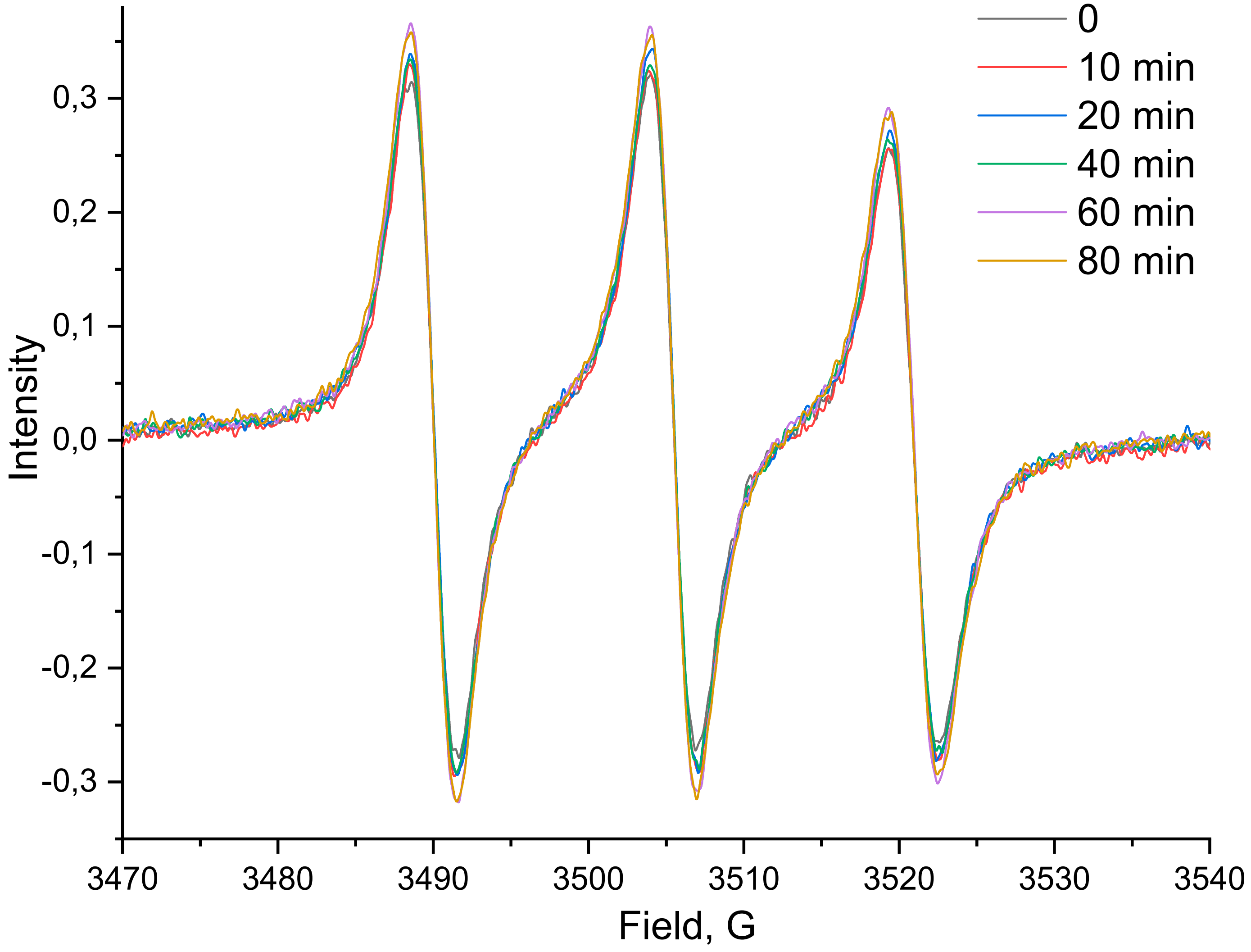

Figure S18. ESR Spectra of compound **10a** in D_2O over time

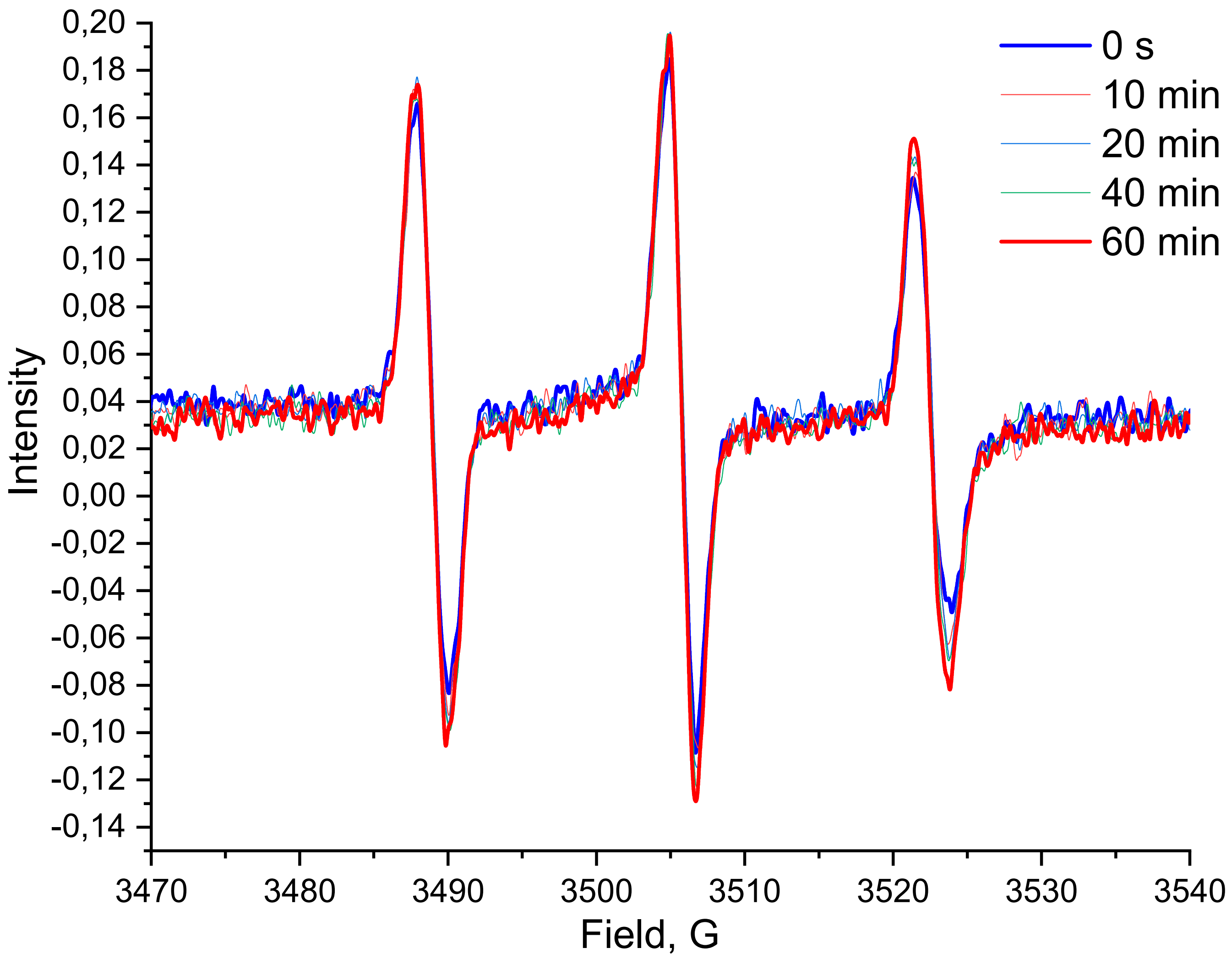

Figure S19. ESR Spectra of compound **10a** in deionized water over time

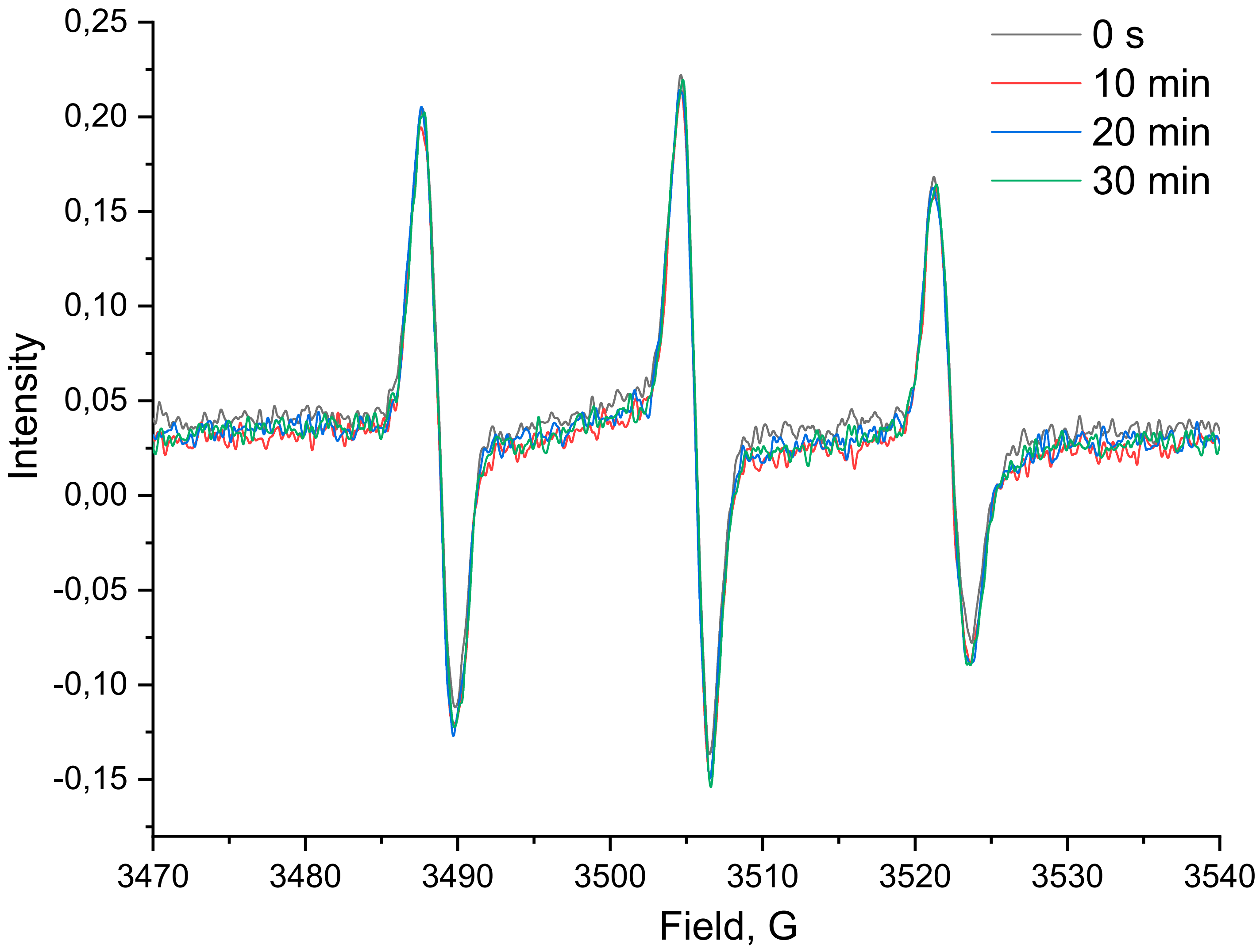

Supplement: Supplementary file 1 [file molecules-27-08414-s001.zip › molecules-2043665-supplementary.pdf]
